# Cerebrospinal Fluid miRNA Profiling as a Potential Liquid Biopsy for Vestibular Schwannomas

**DOI:** 10.3390/ncrna12050035

**Published:** 2026-09-01

**Authors:** Małgorzata Litwiniuk-Kosmala, Maria Makuszewska, Robert Bartoszewicz, Agnieszka Jasińska-Nowacka, Maciej Ołdak, Bartłomiej Gielniewski, Bartosz Wojtaś, Kazimierz Niemczyk

**Affiliations:** 1 Department of Otorhinolaryngology, Head and Neck Surgery, Warsaw Medical University, Banacha 1a st., 02-092 Warsaw, Poland; maria.makuszewska@wum.edu.pl (M.M.); robert.bartoszewicz@wum.edu.pl (R.B.); agnieszka.jasinska@wum.edu.pl (A.J.-N.); kazimierz.niemczyk@wum.edu.pl (K.N.); 2 Laboratory of Sequencing, Nencki Institute of Experimental Biology, 02-093 Warsaw, Poland; m.oldak@nencki.edu.pl (M.O.); b.gielniewski@nencki.edu.pl (B.G.); b.wojtas@nencki.edu.pl (B.W.); 3 Doctoral School of Molecular Medicine, Medical University of Lodz, 90-647 Lodz, Poland

**Keywords:** vestibular schwannoma, CSF, liquid biopsy, miRNA

## Abstract

**Background/Objectives**: This study aimed to identify a characteristic miRNA expression profile in the CSF of patients diagnosed with vestibular schwannoma and evaluate its potential for tumor assessment. **Methods**: In this prospective study, 17 CSF and corresponding tumor samples (seven small tumors—SVS and 10 large tumors—LVS) were collected from patients operated on for VS in a Tertiary Academic Center. The miRNA expression was analyzed using high-throughput RNA sequencing (NovaSeq 6000 Illumina). Data were normalized, and a comparative analysis of miRNA expression rankings was performed between VS patients and a public healthy donor dataset. Functional implications were explored using KEGG pathway enrichment analysis. **Results**: A total of 1633 miRNAs were identified in all CSF samples derived from VS patients. Comparison with healthy donors revealed a moderate ranking correlation (ρ = 0.39), with significant shifts for specific molecules like hsa-miR-766-3p and hsa-miR-182-5p. Only six miRNAs were found to correlate between CSF and tumor tissue, while 16 exhibited a negative correlation. No statistical correlation was found between tumor size and the CSF miRNA profile. KEGG analysis highlighted enriched pathways, including neurotrophin signaling and focal adhesion. **Conclusions**: The results of our study support the feasibility of miRNA-based CSF liquid biopsy for VS assessment. However, the results of miRNA expression profiling conducted in tumor tissue cannot be directly transferred into CSF sample analyses. Further studies are warranted to explain this phenomenon and to search for reliable miRNA markers of VS progression in the CSF liquid biopsy specimens.

## 1. Introduction

Vestibular schwannomas (VS) are tumors that develop from myelin-producing Schwann cells surrounding branches of the vestibulocochlear nerve [1]. Despite their benign nature, they remain a major clinical challenge, as they present a varied natural history. In about 50% of patients, these tumors are characterized by very slow or no growth in long-term observation and thus can be managed conservatively, without the need for surgical intervention. At the other extreme, there are more aggressive variants of VS tumors, characterized by rapid growth and causing early hearing loss [2]. Currently, there is an urgent need to identify molecular biomarkers that could help to predict tumor growth rate or identify tumors of a potentially more aggressive type early in the disease. This would allow for the identification of patients who require early invasive treatment. Recently, we identified several types of small non-coding RNAs (microRNAs, miRNAs), that are associated with increased tumor growth and early hearing loss in tumor tissue samples derived from VS patients [3,4]. Nevertheless, a surgical biopsy of an intracranial tumor, such as a VS, might be a complicated, risky and costly procedure. Therefore, the idea of a “liquid biopsy” has emerged in recent years, which means identifying molecular markers of central nervous system (CNS) tumors in cerebrospinal fluid (CSF) [5,6]. Circulating microRNAs have been found in almost all human body fluids, including CSF [7]. Studies have so far been conducted to determine specific miRNA profiles in CSF in various CNS tumors, including gliomas, meningiomas, and brain metastases [8,9,10]. The currently available literature lacks data on the analysis of the CSF miRNA profile in patients with VS. Therefore, the aim of the present study was to identify the CSF miRNA expression profile characteristic of the presence of VS, particularly its aggressive variant.

## 2. Results

### 2.1. Patients Characteristics

General characteristics of the patients included in the study are summarized in Table 1.

### 2.2. Total miRNA Expression in CSF Samples and CSF-Tumor vs. Tissue Correlation

A total of 1633 miRNAs were identified across all CSF samples, while 2226 miRNAs were detected in the corresponding tumor tissue. Following expression-level normalization, 22 miRNAs demonstrated significant correlations between the CSF and tumor profiles. Specifically, 16 miRNAs exhibited a negative correlation, and six showed a positive correlation, as detailed in Table 2.

### 2.3. Comparison of CSF miRNA Profile Between Healthy Donors and VS Patients

**Figure 1:**
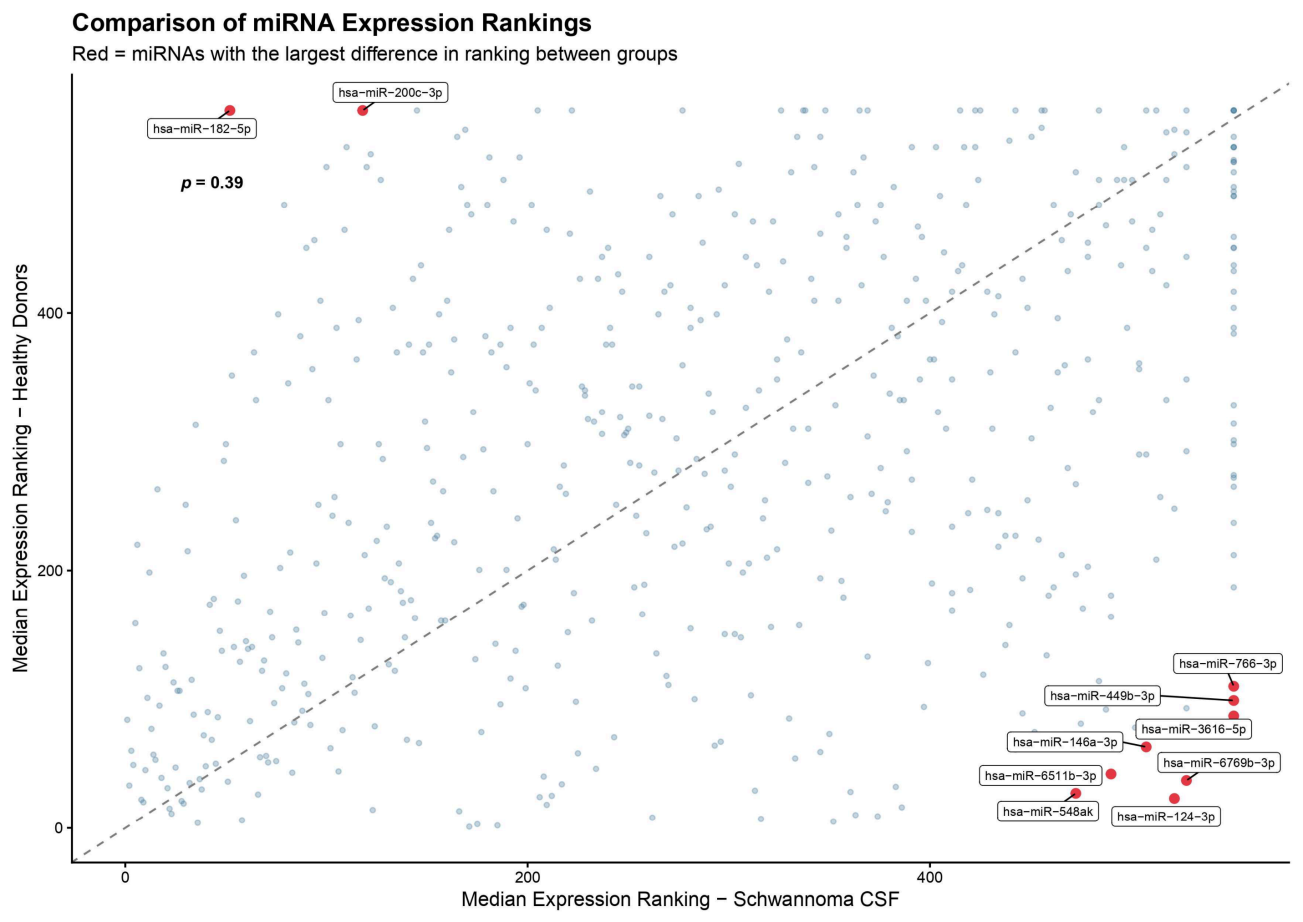
Comparison of miRNA expression rankings between cerebrospinal fluid (CSF) samples from patients with vestibular schwannoma and a publicly available healthy donor dataset. miRNAs with the greatest differences between the groups are highlighted in red.

The miRNA expression profiles of the collected samples were compared with a publicly available dataset of miRNA expression profiles from healthy donors. To account for potential batch effects between the datasets, a comparative analysis of miRNA expression rankings was performed. A global comparison revealed a moderate positive correlation, with a Spearman’s rank correlation coefficient of ρ = 0.39. Despite this general trend, several miRNAs exhibited pronounced shifts in their expression hierarchy. Specifically, a subset of miRNAs—including hsa-miR-766-3p, hsa-miR-449b-3p, hsa-miR-3616-5p, hsa-miR-146a-3p, hsa-miR-6769b-3p, hsa-miR-6511b-3p, hsa-miR-548ak, and hsa-miR-124-3p—showed substantially higher rankings in the Schwannoma CSF group compared to healthy donors. Conversely, hsa-miR-182-5p and hsa-miR-200c-3p were characterized by significantly higher rankings in the healthy donor group, indicating a relative decrease in their expression prominence within the CSF of schwannoma patients. The detailed rank expression comparison is illustrated in Figure 1.

The results of the KEGG pathway enrichment analysis for the differentially expressed miRNAs are summarized in Table 3. The ten most significantly enriched pathways are presented, highlighting the biological processes potentially modulated by the miRNA expression profile identified in the CSF of patients with vestibular schwannoma.

### 2.4. SVS-LVS Differential miRNA Expression in CSF

**Figure 2:**
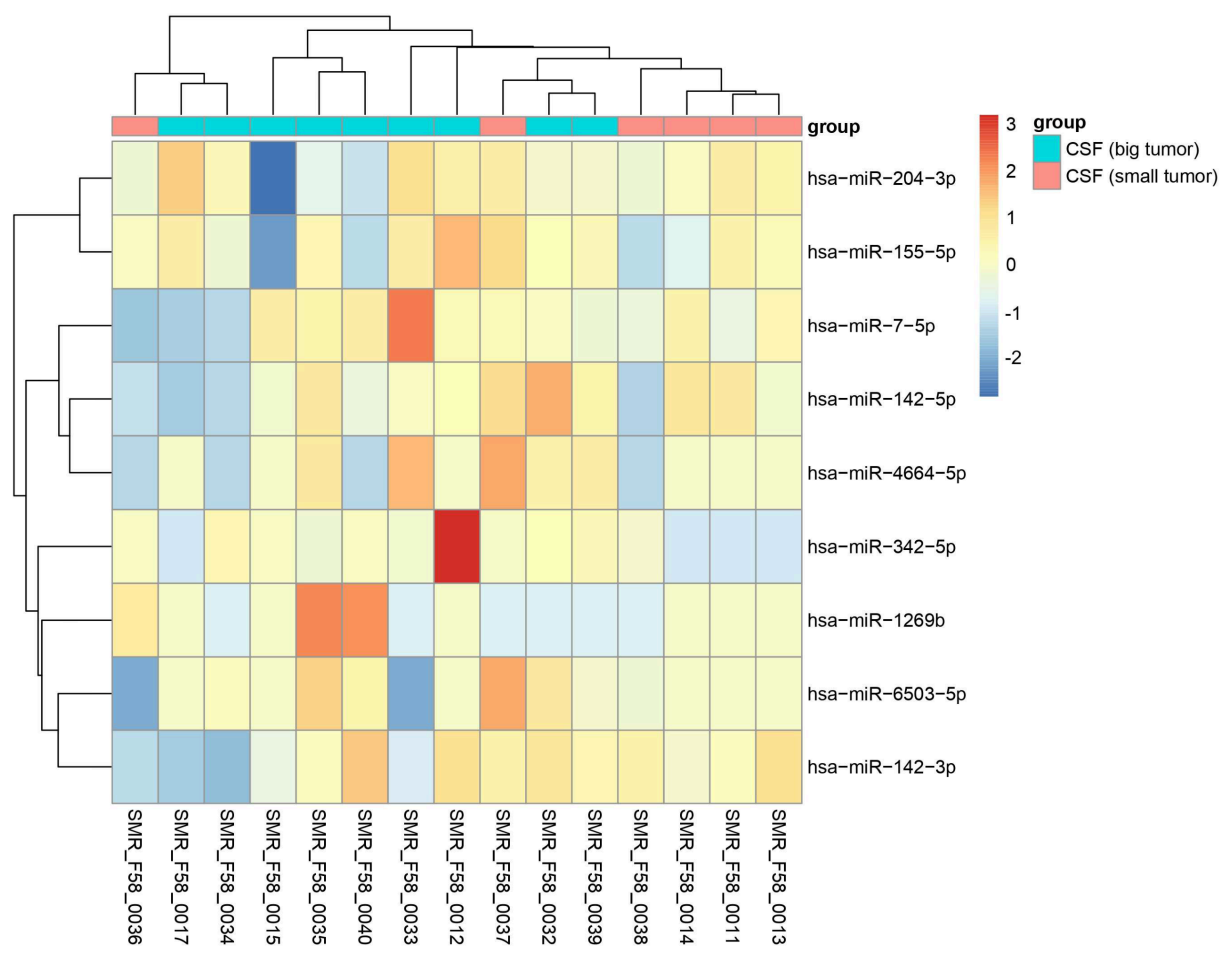
Comparison of cerebrospinal fluid (CSF) miRNA expression profiles between patients with small and large vestibular schwannomas.

The miRNA expression profiles in CSF were compared between patients with small vestibular schwannomas (SVSs) and large vestibular schwannomas (LVSs). To maintain the integrity of the cohort, patients with a history of prior radiotherapy were excluded from this analysis. Statistical evaluation revealed no significant correlation between tumor size and the miRNA expression profile within the CSF. These results are illustrated in Figure 2.

## 3. Discussion

In this study, we conducted a comprehensive analysis of miRNA expression in cerebrospinal fluid (CSF) samples from patients with vestibular schwannoma (VS) using next-generation sequencing (NGS) to characterize their molecular profile. Our findings identified specifically altered miRNAs when compared to healthy donors, which enabled the analysis of their potential biological functions. To our knowledge, this is the first report to characterize the miRNA expression profile in the CSF of patients with VS.

The quest for non-invasive and sensitive methods to diagnose and monitor neurological disorders, particularly central nervous system (CNS) tumors, has driven significant interest in the concept of the “liquid biopsy” [11]. A major challenge in applying liquid biopsy to CNS tumors, however, is the blood–brain barrier (BBB), which severely limits the transport of tumor-derived materials, such as cell-free DNA (cfDNA) and proteins, into plasma or serum [6]. This limitation is largely overcome by sampling the CSF. Studies have shown a greater than 10-fold enrichment in the quantity of tumor-derived DNA in the CSF (CSF-tDNA) compared with plasma [12]. Despite these encouraging initial findings, researchers emphasize the urgent need for standardized collection and storage protocols to obtain high-quality biological material, which is especially necessary for analyses based on nucleic acid sequencing technology [6]. Teunissen et al. [13] suggested a standardized protocol for CSF sample collection and banking; however, this has not been universally adopted. In our study, we used an RNA preservative, as well as RNA extraction kit that was initially designed for urine samples but has been previously successfully used for CSF sample handling as well [10]. Using this spin-column chromatography method of RNA extraction, we were able to obtain a sufficient amount of total RNA for further miRNA sequencing from a total volume of 1 mL of CSF.

In our previous study, we identified several miRNAs that were overexpressed in tumor tissue samples derived from large vestibular schwannoma tumors. We analyzed whether these miRNA expression profiles were reproducible in the corresponding CSF samples. Among all of the miRNAs detected in the CSF samples, we were able to identify only six miRNAs that were positively correlated with tissue samples. Surprisingly, there were 16 miRNAs in the CSF samples that correlated negatively with tumor tissue samples. When Zajdel et al. [14] analyzed the miRNA expression profile in CSF and tumor tissue in DLBCL patients, they observed a similar discrepancy between CSF and tissue profiles. Konishi et al. [15] also reported an inverse correlation between the CSF and tumor tissue samples in a series of gastric cancer patients. Various hypotheses have been proposed to explain this phenomenon. First of all, miRNAs detected in schwannoma tissue samples originate mainly from tumor cells, whereas CSF miRNAs may also derive from other cells involved in tumor microenvironment formation [16]. One of the common features of the tumor microenvironment of nearly all solid tumors is hypoxia. Several miRNAs, miR-22, miR-138 and miR-338, that were negatively correlated between CSF and tumor tissue in our study are known as hypoxia-regulating miRNAs. They play an important role in the tumor microenvironment, regulating cell proliferation, metabolism, invasion and migration in hypoxic conditions [17]. If a miRNA is highly expressed in infiltrating macrophages or stromal cells to suppress local inflammation or promote tissue remodeling but is not actively secreted by these cells into the CSF, its bulk tissue signal will be high while its CSF level remains low. Alternatively, surrounding non-neoplastic cells in the cerebellopontine angle (e.g., compressed cranial nerve VIII fibers or healthy Schwann cells) may secrete specific miRNAs into the CSF as a protective response to a hypoxic microenvironment and mechanical compression. This would result in elevated CSF levels despite low expression in the tumor tissue itself.

Another hypothesis is the selective secretion of miRNAs by tumor tissue, which was observed in brain biopsies and CSF exosomes derived from healthy donors [18]. We believe that miR-431 is the most interesting miRNA in this context. Fujita et al. [19] observed that miR-431 was selectively secreted by a VS tumor in extracellular vesicles and contributed to VS-associated hearing loss following cochlear stress. Taking all these arguments together, one may speculate that miRNA levels detected in the CSF reflect a systemic response to the disease rather than deregulations in the brain lesion itself [14].

The comparative analysis of miRNA expression rankings in the CSF between VS patients and healthy donors revealed a subset of ten miRNAs that were differentially ranked in these groups. The functional implications of the differentially ranked miRNAs were further explored through KEGG pathway enrichment analysis. The results, summarized in Table 3, highlight the ten most significantly enriched biological pathways potentially modulated by the miRNA profile characteristic of the CSF in patients with vestibular schwannoma. These pathways are primarily involved in signal transduction, oncogenesis, and cellular structural organization, including the neurotrophin signaling pathway, pathways in cancer, and focal adhesion. The enrichment of these terms suggests that the identified miRNA signatures—which include molecules with substantial ranking shifts between patients and healthy donors, such as hsa-miR-766-3p and hsa-miR-182-5p, may play a key role in the molecular pathogenesis of vestibular schwannoma. Importantly, there was no overlap between the miRNAs showing a significant correlation between VS tissue and matched CSF and those displaying the greatest ranking differences between VS and healthy donor CSF. This finding suggests that these two analytical approaches may capture distinct aspects of the CSF miRNA landscape. While tissue–CSF correlations may reflect miRNAs more closely associated with tumor-derived molecular processes or their selective release into the CSF, the differences observed between VS and healthy donors may represent a broader disease-associated signature influenced by the tumor microenvironment and other cellular sources within the CSF.

The comparative analysis of miRNA CSF profiles between samples derived from large vestibular schwannoma (LVS) and small vestibular schwannoma (SVS) revealed no specific miRNAs that were differentially expressed between these two patient cohorts. In recent years, various molecular biomarkers of VS growth in the tumor tissue, CSF, and blood have been investigated [20,21]. Specifically, within the CSF, previous studies have noted that elevated concentrations of immunomodulatory cytokines, such as CCL2 and CCL18, are associated with larger tumor volumes [22]. Furthermore, protein-based investigations by Huang et al. [23] identified specific signatures linked to tumor progression, where ABCA3 and KLF11 levels positively correlated with the size of early-stage VS, while BASP1 and PRDX2 showed a negative correlation. The lack of statistically significant findings in our analysis is likely attributable to the relatively small sample size (n = 17), which remains a primary limiting factor in the current study.

This study has several limitations that should be acknowledged and underscore the exploratory and hypothesis-generating nature of our findings. First, it was designed as an exploratory investigation with the primary objective of identifying a characteristic miRNA signature within the CSF of VS patients from a broad pool of miRNAs. Consequently, secondary validation through qPCR or in-depth functional analyses of the specifically altered miRNAs was not performed at this stage. Second, the study cohort was relatively small, consisting of 17 patients (seven with SVS and 10 with LVS tumors), which may affect the statistical power of the findings. Furthermore, the lack of a dedicated internal control group required the use of a publicly available dataset for comparison with healthy donors, which necessitated the use of ranking analyses to mitigate potential batch effects. As CSF collection from healthy individuals was not feasible or ethically justified in our clinical setting, we used a publicly available CSF miRNA dataset as an external reference. Given the limited availability of CSF miRNA sequencing datasets from truly healthy individuals, the selected public dataset represented one of the few suitable references available for comparison and was therefore considered the most appropriate external control. Its sample size (n = 15) was also considered adequate for an exploratory comparison with our VS cohort. Importantly, both datasets were generated using Illumina sequencing platforms (NovaSeq 6000 and NovaSeq 500), providing substantial technical compatibility.

Given the exploratory scope of this proof-of-concept study, future validation efforts must be strategically prioritized to translate these findings into clinically useful biomarkers. Future validation should first target miRNAs exhibiting the most pronounced shifts in expression hierarchy between VS patients and healthy controls. In particular, hsa-miR-766-3p and hsa-miR-182-5p, which showed the greatest discordance in ranking, are prime candidates for RT-qPCR validation. Candidates showing strong negative correlations with tumor tissue—such as the hypoxia-regulated hsa-miR-22-3p, hsa-miR-138-5p, and hsa-miR-338-3p—should be validated to elucidate the host-tumor microenvironment interaction. Finally, hsa-miR-431, which is implicated in selective exosomal secretion and VS-associated hearing loss, represents a crucial target for further validation, taking into consideration its prognostic and therapeutic potential. Furthermore, future studies might incorporate longitudinal CSF sampling (pre- and post-operative) to demonstrate whether successful tumor resection leads to the normalization of these biomarker levels, thereby establishing a definitive causal link between VS presence and CSF miRNA profiles.

## 4. Materials and Methods

### 4.1. Study Protocol

A total number of 20 participants were recruited for this prospective study among patients operated on for VS in the Otolaryngology, Head and Neck Surgery Department at the Warsaw Medical University, a Tertiary Academic Center. CSF and corresponding tumor samples were collected intraoperatively from each patient. All patients signed informed consent to release CSF and tumor tissue samples for research purposes. The study was approved by the local Ethics Committee and was conducted in accordance with the Declaration of Helsinki. Tumor size was obtained from the most immediate preoperative MRI scan and was calculated using AAO-HNS criteria. All tumors limited to the internal auditory canal, with a maximum size of 12 mm, were considered small tumors (SVS), whereas tumors more than 12 mm in size with extracanalicular extension were classified as large, clinically advanced tumors (LVS). Three patients were excluded from the final analysis after obtaining the final histopathological report, since tumor types other than VS were identified. Two patients who underwent radiotherapy (Gamma Knife treatment) prior to surgery were not excluded from the study; however, these samples were marked for further analyses. Ultimately, 17 CSF and corresponding tumor samples (seven SVS and 10 LVS) qualified for further analysis.

The CSF samples were collected from the cerebellopontine angle cistern, immediately after dural opening, using a peripheral venous catheter attached to a syringe. Approximately 1 mL of CSF was transferred directly to a Urine Collection and Preservation Tube (Norgen Biotek, Thorold, ON, Canada), where it was mixed with the urine preservative and stored at room temperature for further analysis. Tumor samples were collected during the microsurgical procedure of tumor removal. All tumor samples were immediately immersed in RNA later, according to the manufacturer’s protocol, and frozen at −20 °C for further analysis.

### 4.2. Total RNA Isolation

A standard Trizol RNA isolation protocol was used to isolate total RNA from tumor tissue samples, as described in detail in our previous paper. A spin-column chromatography method was used to isolate total RNA, including miRNA, from the CSF samples. Prior to RNA extraction, samples were centrifuged at 3000× *g* for 10 min to remove cellular debris. The supernatant was transferred to a new RNase-free tube for RNA isolation. Total RNA was extracted from 1 mL of CSF using the Urine microRNA Purification Kit (Norgen Biotek, Thorold, ON, Canada), following the manufacturer’s protocol. Briefly, 1 mL of CSF was mixed with an equal volume of Lysis Solution A, followed by incubation at room temperature for 10 min to ensure complete lysis. Proteinase K solution was then added, and the sample was incubated at 55 °C for 15 min. Following lysis, ethanol was added to the lysate to facilitate RNA binding to the provided spin column. The lysate mixture was then applied to the purification column and centrifuged at 6000× *g* for 1 min. The flow-through was discarded, and the column was washed sequentially with the provided Wash Solutions A and B, according to the protocol. To remove any residual contaminants, a final centrifugation step was performed. RNA was eluted in 50 µL of Elution Solution A. The eluted RNA was immediately stored at −80 °C until further analysis.

### 4.3. miRNA Sequencing

The integrity of total RNA was assessed using an Agilent 2100 Bioanalyzer with an RNA 6000 Nano Kit (Agilent Technologies, Ltd., Santa Clara, CA, USA). Afterwards, miRNA libraries were prepared using the Qiaseq miRNA Library Kit according to the manufacturer’s protocol (Qiagen, Hilden, Germany). Libraries were evaluated with an Agilent 2100 Bioanalyzer (Agilent Technologies, Ltd., Santa Clara, CA, USA) and subsequently quantified with a Quantus fluorometer (Promega, Madison, WI, USA). Single-end sequencing of libraries was performed using NovaSeq 6000 (Illumina, San Diego, CA, USA). Approximately 20 M reads per miRNA-seq sample were obtained in order to achieve a high probability of finding novel miRNA biomarkers.

### 4.4. Bioinformatics and Statistical Analysis

The initial analysis was performed using the GeneGlobe Qiagen tool(Venlo, Netherlands), where single-end sequencing reads were mapped to the human miRbase database. Unique Molecular Indices (UMI) were counted for all mature small RNA sequences (miRNA, piRNA). The statistical analysis was performed using the R software (4.5.0 versionR Core Team, New Zeland) where UMI counts were normalized using DESeq2, and differential small RNA analysis was performed using DESeq2. Subsequent visualizations were also performed using the R software tool.

To characterize the miRNA landscape in VS CSF and compare it with healthy controls, two independent datasets were analyzed. The VS cohort consisted of CSF samples collected from 17 patients with confirmed vestibular schwannoma. Healthy donor CSF miRNA sequencing data were obtained from the Gene Expression Omnibus (GEO) public repository (series: GSE150174). ([dataset] (2020). miRNA Expression from Cerebrospinal Fluid (CSF) from Prodromal Huntington Disease Patients, geo, V1. https://www.ncbi.nlm.nih.gov/geo/query/acc.cgi?acc=GSE150174, accessed on 10 March 2026.

Given that the two datasets were generated in different laboratories using distinct library preparation protocols and sequencing platforms, a direct statistical comparison of expression values was not performed. Instead, each dataset was processed independently to minimize the influence of technical batch effects.

Raw count matrices from both cohorts were filtered to retain only miRNAs with a minimum of 10 counts in at least two samples within the respective dataset. Library size differences were corrected using the Trimmed Mean of M-values (TMM) normalization method, as implemented in the edgeR package (version 4.8.2). Normalized counts were subsequently transformed to log2-counts per million (log2-CPM) with a prior count of 1 to stabilize variance at low expression levels. Only miRNAs present in both datasets after independent filtering were retained for comparative analyses, resulting in a final set of shared miRNAs.

To quantitatively assess the concordance of miRNA expression tendencies between the schwannoma and healthy donor cohorts, a rank-based approach was employed. For each dataset, miRNAs were ranked by their median log2-CPM expression across all samples in descending order. Spearman’s rank correlation coefficient (ρ) was calculated between the two ranked lists to evaluate the overall agreement in expression ordering between cohorts. The ten miRNAs exhibiting the greatest discordance—defined as the largest absolute difference in rank position between the two groups—were identified as candidates with potentially disease-associated expression patterns and visualized in a scatter plot of ranks.

### 4.5. Functional KEGG Pathway Analysis

To identify the cellular signaling pathways associated with the differentially expressed miRNAs, functional enrichment analysis was performed using the miRSystem web-based tool (available at: http://mirsystem.cgm.ntu.edu.tw/index.php, accessed on 2 May 2026) [24]. This platform enables the integrated annotation of miRNA signatures to their molecular targets, facilitating the identification of potentially modulated biological pathways as categorized by the Kyoto Encyclopedia of Genes and Genomes (KEGG) database.

## 5. Conclusions

This study represents the first comprehensive, exploratory characterization of the miRNA expression profile in the cerebrospinal fluid (CSF) of patients with vestibular schwannoma (VS) using next-generation sequencing (NGS). We demonstrated that the miRNA profile in the CSF of VS patients differs significantly from that of healthy donors. While our findings support the concept of a miRNA-based liquid biopsy as a feasible method for VS tumor assessment, given the preliminary and hypothesis-generating nature of these results, they must be interpreted with caution. The observed discrepancy between expression levels in tumor tissue and CSF suggests that the circulating miRNA profile represents a complex combination of tumor shedding and a broader systemic response. Future multi-center studies utilizing the prioritized validation framework outlined herein in larger cohorts with longitudinal follow-up are required to translate these exploratory candidates into clinically validated biomarkers for VS diagnosis and progression monitoring. The lack of a statistical correlation between tumor size and the CSF miRNA profile underscores the necessity for further research to identify reliable markers of VS progression and to fully elucidate the biological mechanisms driving these differences.

## Figures and Tables

**Table 1:** Clinical characteristics of the patients operated on for vestibular schwannoma.

	Patients (*n* = 17)
**Gender**	
Female	9
Male	8
**Age at surgery**	
Mean	44.5
min	22
max	69
**Tumor size**	
Large tumor	10
Small tumor	7

**Table 2:** Correlation of miRNA expression between tumor tissue and cerebrospinal fluid (CSF) in patients with vestibular schwannoma.

	miRNA	Adjusted *p*-Value		miRNA	Adjusted *p*-Value
**Positive correlation between CSF and tissue**	hsa-miR-5002-5p	4.996 × 10^−7^	**Negative correlation between CSF and tissue**	hsa-miR-338-3p	4.5348 × 10^−5^
	hsa-miR-3181	6.119 × 10^−6^		hsa-miR-765	7.4937 × 10^−5^
	hsa-miR-3937	3.217 × 10^−5^		hsa-miR-7156-3p	0.0001
	hsa-miR-578	0.0001		hsa-miR-6842-5p	0.0002
	hsa-miR-206	0.0003		hsa-miR-370-5p	0.0003
	hsa-miR-188-3p	0.0009		hsa-miR-338-5p	0.0003
				hsa-miR-6730-3p	0.0005
				hsa-miR-4521	0.0006
				hsa-miR-6756-3p	0.0011
				hsa-miR-431-3p	0.0007
				hsa-miR-376a-3p	0.0008
				hsa-miR-3609	0.0008
				hsa-miR-3194-3p	0.0011
				hsa-miR-22-3p	0.0009
				hsa-miR-138-5p	0.0011
				hsa-miR-1302	0.0012

**Table 3:** KEGG pathway enrichment analysis of targets for differentially expressed miRNAs in the CSF of patients with vestibular schwannoma.

Term	Union Targets in the Term	Union miRNAs in the Term
Neurotrophin signaling pathway	48	4
Pathways in cancer	89	4
Focal Adhesion	65	4
Regulation of Actin Cytoskeleton	67	4
Axon Guidance	47	4
MAPK Signaling Pathway	70	4
Insulin Signaling Pathway	70	4
TGF beta Signaling Pathway	29	4
ERBB Signaling Pathway	31	3
WNT Signaling Pathway	34	4

## Data Availability

The datasets generated and analyzed during the current study are available from the public repository Figshare (London, UK) under the link: https://doi.org/10.6084/m9.figshare.24466999 accessed on 2 November 2023). Additional data is available from the corresponding author on reasonable request.

## Abbreviations

The following abbreviations are used in this manuscript:

VS	vestibular schwannoma
SVS	small vestibular schwannoma
LVS	large vestibular schwannoma
CNS	central nervous system
CSF	cerebrospinal fluid
miRNA	microRNA
NGS	next-generation sequencing
cf-DNA	cell-free DNA
CSF-tDNA	tumor-derived DNA in the CSF
qPCR	quantitative polymerase chain reaction
